# A constellation of eye-tracking measures reveals social attention differences in ASD and the broad autism phenotype

**DOI:** 10.1186/s13229-022-00490-w

**Published:** 2022-05-04

**Authors:** Kritika Nayar, Frederick Shic, Molly Winston, Molly Losh

**Affiliations:** 1 Neurodevelopmental Disabilities Lab, Roxelyn and Richard Pepper, Department of Communication Sciences and Disorders, Northwestern University, Evanston, IL USA; 2 Center for Child Health, Behavior and Development, Seattle Children’s Research Institute, Seattle, WA USA; 3 Department of Pediatrics, University of Washington School of Medicine, Seattle, WA USA

**Keywords:** Eye tracking, Autism spectrum disorder, Broad autism phenotype, Social attention, Visual processing, Endophenotype

## Abstract

**Background:**

Social attention differences, expressed through gaze patterns, have been documented in autism spectrum disorder (ASD), with subtle differences also reported among first-degree relatives, suggesting a shared genetic link. Findings have mostly been derived from standard eye-tracking methods (total fixation count or total fixation duration). Given the dynamics of visual attention, these standard methods may obscure subtle, yet core, differences in visual attention mechanisms, particularly those presenting sub-clinically. This study applied a constellation of eye-tracking analyses to gaze data from individuals with ASD and their parents.

**Methods:**

This study included *n* = 156 participants across groups, including ASD (*n* = 24) and control (*n* = 32) groups, and parents of individuals with ASD (*n* = 61) and control parents (*n* = 39). A complex scene with social/non-social elements was displayed and gaze tracked via an eye tracker. Eleven analytic methods from the following categories were analyzed: (1) standard variables, (2) temporal dynamics (e.g., gaze over time), (3) fixation patterns (e.g., perseverative or regressive fixations), (4) first fixations, and (5) distribution patterns. MANOVAs, growth curve analyses, and Chi-squared tests were applied to examine group differences. Finally, group differences were examined on component scores derived from a principal component analysis (PCA) that reduced variables to distinct dimensions.

**Results:**

No group differences emerged among standard, first fixation, and distribution pattern variables. Both the ASD and ASD parent groups demonstrated on average reduced social attention over time and atypical perseverative fixations. Lower social attention factor scores derived from PCA strongly differentiated the ASD and ASD parent groups from controls, with parent findings driven by the subset of parents demonstrating the broad autism phenotype.

**Limitations:**

To generalize these findings, larger sample sizes, extended viewing contexts (e.g., dynamic stimuli), and even more eye-tracking analytical methods are needed.

**Conclusions:**

Fixations over time and perseverative fixations differentiated ASD and the ASD parent groups from controls, with the PCA most robustly capturing social attention differences. Findings highlight their methodological utility in studies of the (broad) autism spectrum to capture nuanced visual attention differences that may relate to clinical symptoms in ASD, and reflect genetic liability in clinically unaffected relatives. This proof-of-concept study may inform future studies using eye tracking across populations where social attention is impacted.

**Supplementary Information:**

The online version contains supplementary material available at 10.1186/s13229-022-00490-w.

## Introduction

The study of eye movement patterns has proven to be a powerful tool for revealing meaningful information about perceptual and attentional strategies, including providing an indirect measurement of underlying cognitive, attentional, and executive skills [1]. Early eye-tracking studies [2], along with many that followed [1, 3–6], have demonstrated that the location of gaze (i.e., where individuals look when visually exploring stimuli) not only reflects attentional processes [7, 8], but also maps onto underlying thoughts and cognition [1]. In this way, eye movement may be considered as both complementary to and associated with both psychophysical (e.g., accuracy and reaction time indices) and neural measurements of cognition [9]. For example, studies have demonstrated that saccade speed or saccade target location predicts reaction time [10, 11], and that analyses of gaze could index activity in areas of the brain associated with visual perception (e.g., visual examination of social stimuli activates the amygdala, and face perception activates the Fusiform Face Area) [12–16]. Eye-tracking also has the potential to reveal moment-to-moment information of underlying cognition, revealing nuanced and dynamic patterns at an individual or group level. Given the often automatic and rapid nature of eye movements, analysis of gaze may thus represent a phenomenon existing as an intermediate link between brain and behavior, with the potential of revealing cognitive differences that may stem from underlying neurobiology.

The use of eye tracking has been particularly revealing in studies of social cognition in autism spectrum disorder (ASD) [17–19]. Specifically, differences in visual attention have been repeatedly documented in individuals ASD relative to controls [20–22], with such differences relating to social communication impairments [17, 23–26] and restricted and repetitive behaviors [27, 28], core features of the disorder. Gaze and eye movement differences can be detected as early as infancy in individuals with ASD (such as lacking a preference between looking at faces versus objects, or showing atypical scanning patterns when gazing at faces) [29–36]. These visual attention differences appear to persist into adolescence and adulthood, wherein individuals with ASD have been reported to show slower latencies to orient to social features of the scene [28, 37] and exhibit different scanning patterns [21, 38]; though findings vary depending on the type of stimulus [26, 39]. For example, when there is competing information in the scene (i.e., both salient and non-salient features), individuals with ASD tend to spend more time exploring non-salient aspects of the scene, such as inanimate objects or non-eye regions of facial stimuli [21, 23, 40, 41]. These differences appear to be clinically meaningful, with several studies reporting a link between atypical gaze patterns and reduced social competency [17, 30].

Eye movement patterns appear to be heritable in the general population [42] and across psychiatric disorders [43], suggesting that eye movements and visual attention may not only reflect underlying genetics (i.e., constituting endophenotypes, which are heritable characteristics associated with the genetic underpinnings of a disorder [44]), but also neurobiological mechanisms contributing to the development of a disorder’s symptomatology. Studies of gaze in individuals with ASD and their relatives thus have the potential to inform the mechanistic processes related to the etiology and development of ASD symptomatology, as well as subclinical features of the broad autism phenotype (BAP), which are linked to underlying genetic liability in clinically unaffected relatives [45–47]. Indeed, subtle differences in gaze have been reported among first-degree relatives of individuals with ASD [23, 42, 47, 48], and particularly among those who display the BAP [48–51]. For instance, Adolphs et al. [48], showed that parents who displayed features of the BAP relied less on the eye region of the face when making judgments about emotions. Less fluent gaze-language coordination and subtle differences in gaze when narrating from illustrated stimuli have also been reported among parents, and linked with BAP features [23, 27].

Together, this work demonstrates the utility of studying looking patterns as potentially good candidate endophenotypes, given their known heritability and association with ASD. However, visual attention and related eye movement patterns have typically been examined using primarily global fixation measures across many different stimuli across studies, and have been understudied in first-degree relatives with only three studies to date examining visual perception/attention using eye tracking in parents [23, 27, 51]. Further study is therefore warranted to understand the nature and extent of gaze differences in relatives, how such differences may overlap with those evident in ASD, and important connections to underlying cognitive mechanistic and biological factors. Additionally, evidence suggests that study results dependent on standard fixation measures are heavily influenced by processing methods [52–54], and have been shown to dramatically alter findings in ASD [54]. Finally, these standard measures provide only a global overview of looking patterns, and may miss potentially important subtleties evident in ASD and the BAP.

Ongoing studies have demonstrated the efficacy of applying other methods of analyses to examine eye-tracking data, which may reveal important aspects of cognition. For example, perseverations (repeat successive fixations towards the same area of interest) may reflect “sticky attention” or mental disengagement [55], whereas regressions (i.e., refixating on previously examined areas of interest) may index the loss of mental set or executive control, as the looker’s attention is captured [56–59]. Analysis of both these fixation types have been fruitfully applied in studies of ASD and the BAP using social and non-social stimuli, and were also found to relate to ASD symptomatology [27, 28]. Shic and colleagues [41] utilized another fixation analytic method, transition entropy analyses, in young children with ASD, finding no changes in the transitions of fixations (i.e., transitions from one AOI to another) between salient and non-salient regions of the face in individuals with ASD, unlike controls, thus demonstrating differences in patterns of attention allocation between groups. Spatial Distribution Analysis or Nearest Neighbor Index (i.e., distance-dispersion algorithm) were also examined in children with and without ASD, to explore how fixations were dispersed across the facial stimuli [60]. Finally, growth curve analyses (GCA), proves to be a rigorous method of assessing changes over the course of time, which can be applied to understand the moment-to-moment pattern of gaze while interpreting a scene [61, 62]. In particular, GCA maps out the time-linked gaze trajectory over the course of the stimulus presentation and can elucidate changes occurring longitudinally across developmental time periods, such as those found in infants with and without ASD [63]. As such, while traditional methods of dwell time and fixations can reveal important aspects of information processing, they may lack sensitivity for capturing more nuanced patterns of eye movement that can be revealed through more detailed and expansive methods. Examining temporal dynamics is critical to be able to capture social attentional patterns granularly, thus allowing a vivid illustration of the social attentional trajectories that unfold over time and reflecting the dynamic (versus static) process of social attention in the real world.

The present study was an attempt to address this concern, through application of a suite of eye-tracking analytic tools to gaze data in ASD and the BAP, with the goal of evaluating potentially more sensitive methods of gaze patterns than global analyses of looking time or fixations, and that might help to capture subtle top-down social visual attentional differences in non-clinical populations such as the BAP. Based on evidence previously reviewed and the social atypicalities inherent to ASD, and more subtly in the BAP, it was predicted that individuals with ASD and their parents (particularly those with features of the BAP) would show reduced social attention and increased attention to non-salient components of the scene, reflective of decreased attribution of social salience. Based on evidence demonstrating relationships between perseverative eye movement patterns and restricted and repetitive behaviors, a larger number of refixations (repeated fixations) and shorter first fixation duration was also expected, with decreased spatial distribution of fixations, and atypical fixation transition and looking patterns over the course of the task compared to controls.

## Methods

### Participants (Table 1)

Participants included in the present study were identical to those included in a prior study exploring language and related looking patterns [23]. Twenty-nine individuals with ASD (ASD group) and 34 control participants (control group), as well as 74 parents of individuals with ASD (ASD parent group) and 45 control parents (control parent group) were included in the study. Inclusion criteria for individuals with ASD and controls included being 15 years of age and older and having a Full Scale IQ (FSIQ) and Verbal IQ (VIQ) ≥ 80. Participants were excluded for any severe psychiatric disorder (e.g., schizophrenia, bipolar disorder) and uncorrected vision impairments (e.g., strabismus). Participant characteristics are outlined in Table 1. All procedures were approved by the University’s Institutional Review Board and written informed consent/assent were obtained for all participants.

**Table 1:** Sample characteristics

	Control group			ASD group			Group difference			
	*M*	Range	SD	*M*	Range	SD	*t*	*df*	*p*	Cohen's *d*
**Probands** n (M/F)	**32 (15/17)**			**24 (18/6)**			–	–	–	–
Age (years)	20.90	15–33.25	5.15	23.82	15.19–57.46	9.28	− 1.50	54	0.139	− 0.405
FSIQ	116.0	89–135	12.1	110.3	83–131	12.9	1.70	54	0.095	0.459
VIQ	118.1	93–138	11.9	108.8	84–132	13.1	2.80	54	**0.007**	0.756
PIQ	110.8	79–129	13.1	110.1	68–131	15.1	0.19	54	0.847	0.051
ADOS Total Severity Score^a^	–	–	–	6.1	1–9	2.3	–	–	–	–
SA Severity Score	–	–	–	5.8	2–9	1.9	–	–	–	–
RRB Severity Score	–	–	–	7.3	5–10	1.8	–	–	–	–
ADI-R Algorithm A (communication)	–	–	–	16.7	9–27	6.7	–	–	–	–
ADI-R Algorithm B (social)	–	–	–	14.8	4–23	5.3	–	–	–	–
ADI-R Algorithm C (RRB)	–	–	–	6.0	2–11	2.4	–	–	–	–
Reading the Mind in the Eyes (% correct)	74.94	55.56–91.67	9.33	63.0	30.56–78.57	13.7	3.56	37.53	**0.001**	0.961

	Control parent group			ASD parent group			Group difference			
	*M*	Range	SD	*M*	Range	SD	*t*	*df*	*p*	Cohen's *d*
**Parents n (M/F)**	**39 (17/22)**			**61 (29/32)**			–	–	–	–
Age (years)	40.02	22.94–60.92	9.82	44.93	28.38–63.19	7.33	*− 2.67*	*65.35*	***0.010***	*− 0.547*
FSIQ	115.1	96–136	9.8	111.0	86–136	10.9	1.92	98	0.058	0.394
VIQ	111.4	91–138	12.5	108.5	84–130	10.2	1.26	98	0.210	0.258
PIQ	114.8	91–148	10.3	111.0	77–137	11.9	1.64	98	0.104	0.336

	BAP(−) parent group			BAP(+) parent group						
	*M*	Range	SD	*M*	Range	SD				
**BAP n (M/F)**	**36 (13/23)**			**24 (16/8)**						
Age (years)	42.80	28.38–54.32	6.96	47.80	33.84–63.19	6.90				
FSIQ	109.8	86–134	109.8	112.8	93–136	9.8				
VIQ	107.4	84–130	107.4	110.3	89–126	9.0				
PIQ	110.1	83–131	110.1	112.4	77–137	12.4				

Bold indicates significance *p* < 0.05; Italics indicates unequal variance assumed; ^a^Comparison severity score labels are as follows: 0–2 = “minimal-to-no evidence”, 3–4 = “low”, 5–7 = “moderate”, 8–10 = “high”. ADOS, Autism Diagnostic Observation Scale; FSIQ, Full-Scale IQ; PIQ, Performance IQ; RRB, Restricted and Repetitive Behaviors and Interests; SA, social affect; VIQ, Verbal IQ.

Individuals with ASD were included following confirmation of ASD with gold standard instruments (Autism Diagnostic Observation Schedule-General or 2nd Edition (ADOS) [64] and/or the Autism Diagnostic Interview-Revised (ADI-R) [65]). Parents of an individual with ASD were included if they had at least one child with idiopathic ASD, and control participants were required to have no personal or family history of ASD or related genetic disorders (e.g., fragile X syndrome). BAP status was assessed in the ASD parent group only, given low base rates of the BAP among individuals without a family history of ASD [49, 66, 67]. BAP status was assessed using the Modified Personality Assessment Scale-Revised (MPAS-R) [66], which includes a series of questions specifically designed to tap the subclinical features related to the BAP including aloof, rigid, perfectionistic, and untactful personality traits. Coding of personality features followed methods outlined in prior work, such that raters were assigned scores ranging from 0 to 2 (trait absent, possibly present, definitely present) on a 5-point Likert scale.

### Eye tracking procedures

#### General procedures

Participants were asked to narrate a story after looking at an image being presented for 8 s on a 17-inch TFT LCD (1280 × 1024 resolution) Tobii T60 series eye tracker (Tobii Technology AB, Danderyd, Sweden). All participants were seated 50–60 cm from the screen and had their gaze calibrated prior to task administration, including using a standard 5-point calibration grid, which has a visual angle accuracy of 0.5°. Participants were recalibrated following any large movements. Tracking was monitored live during task administration using Tobii Studio’s built-in live view and track window options, with additional calibration checks embedded in the task (e.g., center crosshair, corner star) to ensure tracking accuracy.

#### Eye tracking task

**Fig. 1:**
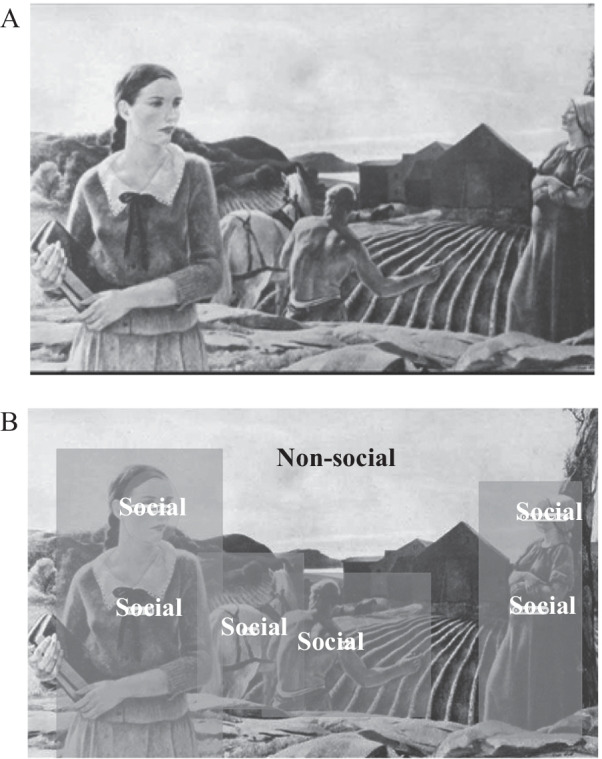
**A** TAT image examined—Card 2; Farmland Scene; **B** Two primary areas of interest (AOIs) were generated—Social AOI, which included all the characters in the image; and Non-social AOI, which included everything else such as the book, barn, field

The thematic apperception test (TAT) [68] was developed as a psychological projective test, and has been used in numerous studies of narrative elicitation [23, 69–72]. Prior work [23] has demonstrated gaze differences in ASD and ASD parent groups when generating stories from TAT stimuli. The current study focused on the “Farmland Scene” (greyscale card 2; Fig. 1A) from the TAT, because of its complexity and prior findings that global indices of fixation revealed differences in attention to setting and protagonists among individuals with ASD and their parents with the BAP [23].

#### Data processing

*Areas of Interests (AOIs).* AOIs were manually drawn in Tobii Studio. The AOIs in the Farmland Scene are depicted in Fig. 1B, and were categorized as either social (e.g., human figures) or non-social (e.g., barn in the background). “Buffer” regions were created identical to prior work, such that each AOI was proportionally expanded by up to 10% of its original size [73]. When social and non-social AOIs overlapped, the final AOI designation was assigned as social.

*Gaze processing parameters.* Eye movements were recorded for both eyes with a sampling rate of 60 Hz. Parameters to account for data loss in working with populations with neurodevelopmental disorders were modeled in line with previous pipelines [53]. These parameters were consistent with prior work [23] and the built-in I-VT fixation filter in Tobii Studio as follows—(1) fixations were based on the strict average across both eyes; (2) a velocity threshold of 35 degrees per second was established; (3) adjacent fixations were merged if fixations were less than 100 ms apart and angles were less than 0.5 degrees apart; (4) missing data were linearly interpolated based on a 150 ms maximum continuous gap; and (5) noise reduction was addressed by utilizing a moving average window of 3 samples. Finally, following data export, fixations were set to be a minimum duration of 100 ms (i.e., fixation durations less than 100 ms were excluded).

*Quality control procedures.* Track loss was based on prior work such that participants’ data were excluded if their overall fixation count on the Farmland Scene was < 5 and the total fixation duration was < 4 s (i.e., gaze data was reliably collected for at least half of the 8 s that the stimulus was presented). These criteria resulted in the exclusion of 17% ASD (*n* = 5), 6% control (*n* = 2), 18% ASD-parent (*n* = 13) and 13% control parent (*n* = 6) participants’ data. Fisher’s Exact Test (FET) and Chi-Squared Test revealed no significant group differences in the proportion of valid or invalid data in ASD versus controls (FET *p* = 0.233) or in ASD parents versus control parents (*X*^2^(1,119) = 0.374, *p* = 0.541), respectively. The final sample was as follows: *n* = 29 in the ASD group, *n* = 34 in the control group, *n* = 61 in the ASD parent group, and *n* = 39 in the control parent group. From the sample of parents of individuals with ASD, 24 were characterized as BAP(+) (i.e., meeting criteria for having BAP traits), and 37 were characterized as BAP(−) (i.e., not meeting criteria for the BAP).

#### Eye-tracking variables

*Standard gaze variables:* Dwell time and fixation count were included in an existing study (Lee et al., 2019), and re-reported here for the purpose of examining the efficacy of more nuanced eye-tracking variables in relation to the overall gaze variables. The below metrics are thought to reflect attentional engagement as well as processing time [1].

**(1)** **Dwell time**—Percentage of looking time (sec) toward an AOI was derived by summing the fixation duration of each AOI and dividing it by the total duration of looking, multiplied by 100.

**(2)** **Fixation count**—Percentage of the number of fixations was captured by summing the total number of fixations toward an AOI out of the total number of fixations across the duration of stimulus presentation, multiplied by 100.

##### Temporal dynamics

**(3)** **Fixations over time**—Growth curve analyses (GCA) were employed to investigate change in looking patterns (percentage of fixations) over the course of the stimulus presentation towards social versus non-social AOIs, adapted from the *EyetrackingR* package [74]. To account for track loss at the end of the image as well as pre-established attention-capturing stimuli (i.e., center and corner crosshairs) prior to stimulus presentation, 7 s of the 8 s image were examined (500 ms removed from the beginning and end of the stimuli), using 1 s time bins. Divergence analyses were then applied to examine at which point(s) groups differed from one another in terms of social versus non-social looking. Divergence analyses applied t-tests that are embedded within the *divergence vignette* from the *EyetrackingR* package [74]. Time bins were set to 300 ms to match the approximate average fixation duration across participants. Taken together, these sets of analyses not only provide information on the dynamic patterns of looking via GCA, but also delineate the exact moments in which groups diverge from one another.

##### Fixation patterns

**(4)** **Perseverative fixations**—Percentage of perseverative fixations were derived by summing fixations that occurred in succession toward the same AOI, divided by the total number of fixations, multiplied by 100. Perseverative fixations are thought to tap into attentional “stickiness” or mental disengagement [55].

**(5) Regressive fixations**—Percentage of regressive fixations was captured as the percentage of times a participant returned their gaze to a specific AOI that had already been previously explored, by summing the number of fixations that occurred towards an AOI previously fixated (not including successive fixations/perseverative fixations), divided by the total number of fixations, multiplied by 100. Regressions are thought to reflect slower processing speed or the loss of mental set/executive control [56–59], and reflects the information that repeatedly attracted the viewer’s attention.

**(6)** **Fixation transition analysis**—Examining transitions between different AOIs provides an estimate of general exploration. For the purpose of this study, to demonstrate the utility of examining transitions, we explored the transitions between social and non-social information in four ways: (i) social to social AOI transitions, (ii) non-social to non-social AOI transitions, (iii) social to non-social AOI transitions, and (iv) non-social to social AOI transitions. Percentages based on the total number of transitions were calculated for (i)–(iv). Fixation transition has been shown to reflect shifts in attention [9], in addition to reasoning abilities through the process of comparison between task relevant and task irrelevant information [75–77].

##### First fixations

**(7)** **First fixation AOI**—The percentage of first fixations toward social or non-social information was measured by summing the total number of first fixations that was social or non-social and dividing it by the total number of first fixations, multiplied by 100. First fixation AOI is thought to index the utilization of peripheral visual information, that is associated with global or rapid and automatic visual information processing and generally reflects visual information preference [78].

**(8)** **First fixation duration**—The first fixation duration was derived by measuring the time (in sec) spent examining any AOI during the first fixation (i.e., the first fixation that occurs after the stimulus appears) before making a fixation transition. The first fixation duration on an AOI reflects how much either social or non-social information initially attracted the looker’s attention.

##### Distribution patterns

**(9)** **Fixation rate (exploration)**—In order to obtain a general measure of fixation rate (i.e., exploration) regardless of AOI type was examined. The total number of fixations per participant was divided by the total time spent examining the scene, to produce the number of fixations that occurred per second of track time regardless of AOI. A higher number indicates a greater number of fixations occurring per second of track time, reflecting greater exploration or fixation rate.

**(10)** **Fixation rate (exploration) AOI**—To investigate how much participants explored either social or non-social AOIs, the number of fixations per track time (in sec) toward social or non-social information was further explored. Greater exploration reflects a larger number of fixations toward social or non-social information for a given second, and provides a general indication of attentional capacity and cognitive load [79].

**Fig. 2:**
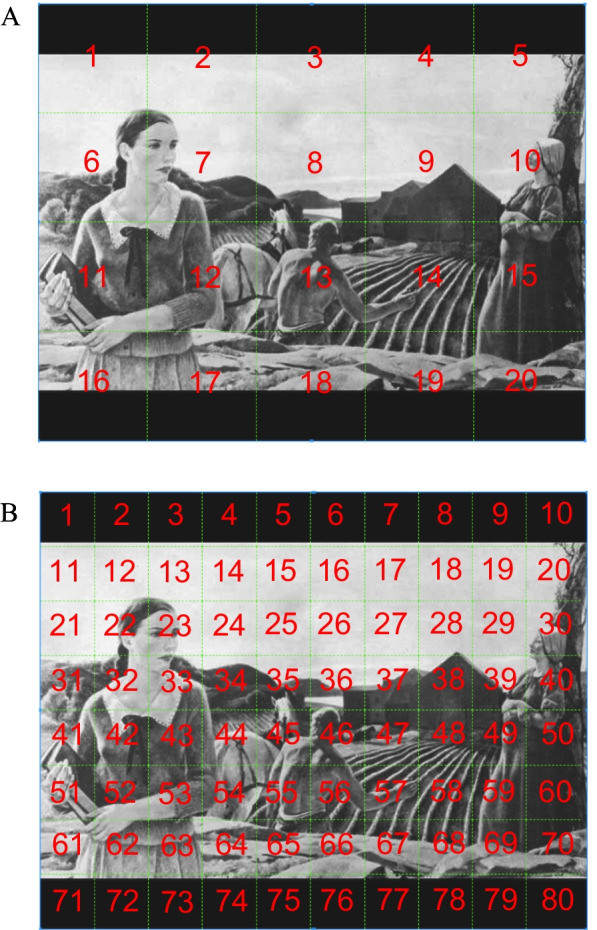
Schematic representing fixation spatial distribution/coverage analysis AOIs. **A** Large areas (5 × 4 grid) and **B** small areas (10 × 8 grid)

**(11)** **Fixation spatial distribution/coverage analyses** (Fig. 2)—Spatial distribution/coverage analyses were conducted to obtain an estimate of how much of the screen was being explored regardless of social or non-social AOI. Given that the Tobii T60 screen was 1280 × 1024 pixels, a 5 × 4 matrix of 20 large areas (256 × 256 pixels/6.45° × 6.45°) and a 10 × 8 matrix of 80 small areas (128 × 128 pixels/3.2° × 3.2°) were generated. Based on prior work [80] showing that the attention maintaining and capturing abilities of an AOI increases with size but asymptotes at 3° visual angle (i.e., 120 × 120 pixels using the T60 Tobii display) for ASD and control groups, the smaller area 10 × 8 matrix would be the most appropriate for the present study while still maintaining equal sized “boxes”. Each fixation point’s location was categorized into one of these 20 or 80 “boxes”, respectively. To account for the different number of fixations per participant, the percentage of mini areas explored was computed per participant by taking the number of areas explored and dividing it by the total number of fixations for that participant, which was then multiplied by 100. This final percent coverage was included in subsequent analyses for larger (5 × 4 matrix) and smaller (10 × 8 matrix) areas. A higher percentage represents greater coverage overall (i.e., a greater proportion of fixations were covering unique areas not previously explored), while a lower percentage indexes less coverage or scatter. This measure indicates whether visual attention was “trapped” within certain general regions of a stimulus, or whether there is greater flexibility in underlying attentional mechanisms.

### Statistical analyses

#### General analytic plan

All analyses were conducted using the following comparisons: (i) ASD group versus control group; (ii) ASD parent group versus control parent group; and (iii) BAP(+) versus BAP(−) versus control parent group, to examine the role of BAP status on overall group differences among parents. Group differences were adjusted using the Benjamini–Hochberg method [81] using a FDR of 0.10 to reduce false negatives and thereby potentially missing important effects [82]. Benjamini–Hochberg adjusted *p* values are reported in the tables; all other *p* values meeting an alpha criterion of < 0.05 are reported below.

#### Assumptions testing

Data were examined to ensure model assumptions of primary statistical tests (i.e., multivariate analysis of variance; MANOVA) were met. Gaze variables for social and non-social looking were included in assumptions testing, and were conducted separately for proband and parent groups. All assumptions were adequate, with the exception of homogeneity of variance–covariance matrices for some variables and sphericity assumptions for others. As such, across all variables, findings using the more robust Pillai’s Trace [83] are reported.

#### Group differences, and growth curve analyses

Group differences in standard gaze variables, fixation patterns, and fixation rates were examined using a one-way MANOVA separately for proband and parent (including by BAP status) groups, with social and non-social AOIs as the dependent variables. Only significant MANOVAs were followed up with univariate ANOVAs. For BAP-level differences, additional planned post-hoc pairwise comparisons (i.e., BAP(+) versus BAP(−) versus control parent groups) were conducted when the overall MANOVA or univariate ANOVAs were significant. Given that the percent of perseverations towards social and non-social information was skewed, MANOVA results were followed up with nonparametric Mann–Whitney U tests. To examine gaze variables not involving social and non-social AOIs (i.e., fixation spatial distribution/coverage, first fixation duration), one-way ANOVAs were conducted separately for proband and parent groups.

Due to their lower sample sizes and categorical nature, first fixation AOIs were analyzed using frequency tests. Sample sizes were low for first fixations towards non-social information (*n* = 6 individuals with ASD/controls and *n* = 17 parents of individuals with ASD/control parents), relative to a greater number of participants who looked towards social information upon their first fixation (*n* = 50 ASD/Controls and *n* = 83 ASD parents/Control parents). As a result, a series of 2 × 2 contingency tables using Fisher’s exact tests were performed separately in parent and proband groups to examine group differences in the proportions of first fixations that were directed towards social and non-social AOI.

To investigate changes in looking patterns towards social versus non-social AOIs over the course of the stimulus presentation, growth curve analyses (GCA) were utilized using similar methods applied in recent work examining gaze during affective facial expressions [84]. Specifically, orthogonal polynomial terms, each representing a different pattern of looking, were added in a stepwise fashion: (1) the linear term reflected an increase or decrease in proportion of looking over time linearly; (2) the quadratic term reflected the dynamic nature of switching from one AOI to another and back again; and (3) the cubic term reflected the timing of switches between AOIs. Only interactions that include a polynomial term were reported.

Finally, a principal component analysis (PCA) was applied to determine whether the large number of gaze variables may be captured by distinct dimensions based on empirical patterns (i.e., principal components), representing most of the information from the original variables [85]. Variables in the PCA included: standard gaze variables (dwell time, fixation count), fixation patterns (perseverative fixations, regressive fixations, fixation transition analyses), first fixation duration, and distribution patterns (fixation rates, fixation spatial distribution/coverage analyses) (see asterisks in Table 2, and Additional file 1: Table S1 for a correlation matrix of all eye-tracking variables). All data from proband and parent groups were included to generate components. Regression factor scores were generated for each component for each individual, and analyzed using one-way ANOVAs to examine group differences among the proband and parent/BAP groups and controls.

**Table 2:** Definitions of eye-tracking variables

Variable	Variable definition
**Standard gaze variables**	
(1) Dwell time*	Percentage of looking time (sec) toward an AOI was derived by summing the fixation duration of each AOI and dividing it by the total duration of looking, multiplied by 100
(2) Fixation count*	Percentage of the number of fixations was captured by summing the total number of fixations toward an AOI out of the total number of fixations across the duration of stimulus presentation, multiplied by 100
**Temporal dynamics**	
(3) Percentage of fixations over time	Growth curve analyses (GCA) were employed to investigate change in looking patterns (percentage of fixations) over the course of the stimulus towards social versus non-social AOIs. To account for track loss at the beginning and end of the stimulus presentation, 7 s of the 8 s image were examined (500 ms removed from the beginning and end of the stimuli), using 1 s time bins. Follow-up analyses examined the divergence between groups of social versus non-social looking using t-tests with 300 ms time bins
**Fixation patterns**	
(4) Perseverative fixations*	Percentage of perseverative fixations were derived by summing fixations that occurred in succession toward the same AOI, divided by the total number of fixations, multiplied by 100
(5) Regressive fixations*	Percentage of regressive fixations was captured as the percentage of times a participant returned their gaze to a specific AOI that had already been previously explored, by summing the number of fixations that occurred towards an AOI previously fixated (not including successive fixations/perseverative fixations), divided by the total number of fixations, multiplied by 100
(6) Fixation transition analysis*	Transitions between social and non-social information were explored in four ways: (i) social to social AOI transitions, (ii) non-social to non-social AOI transitions, (iii) social to non-social AOI transitions, (iv) non-social to social AOI transitions, and (v) total transitions between social and non-social AOIs. Percentages based on the total number of transitions information were calculated for (i)–iv)
**First fixations**	
(7) First fixation AOI	The percentage of first fixations toward social or non-social information was measured by summing the total number of first fixations that was social or non-social and dividing it by the total number of first fixations, multiplied by 100
(8) First fixation duration*	The first fixation duration was derived by measuring the time (in sec) spent examining any AOI during the first fixation (i.e., the first fixation that occurs after the stimulus appears) before making a fixation transition
**Distribution patterns**	
(9) Fixation rate (exploration)*	The total number of fixations per participant was divided by the total time spent examining the scene, to produce the number of fixations that occurred per second of track time regardless of AOI
(10) Fixation rate (exploration) AOI*	The number of fixations per track time (in sec) toward social or non-social information was calculated
(11) Fixation spatial distribution/coverage*	First, a 5 × 4 matrix of 20 large areas (256 × 256 pixels / 6.45° × 6.45°) and a 10 × 8 matrix of 80 small areas (128 × 128 pixels / 3.2° × 3.2°) were generated. Each fixation point’s location was categorized into one of these 20 or 80 “boxes”, respectively. To account for the different number of fixations per participant, the percentage of mini areas explored was computed per participant by taking the number of areas explored and dividing it by the total number of fixations for that participant, which was then multiplied by 100. This final percent coverage was included in subsequent analyses for larger (5 × 4 matrix) and smaller (10 × 8 matrix) areas

*Variables included in the principal component analysis

#### Covariates

Although verbal IQ significantly differed between the ASD and control groups, and full-scale IQ was marginally different between parent groups, IQ was not associated with any eye-tracking outcome variable by group. Additionally, IQ variability is an inherent phenotype of ASD [86], together providing little empirical and conceptual justification for including IQ as a covariate in eye-tracking analyses. Given the large age range of participants included in this study, and that age was significantly associated with several outcome variables for the ASD and ASD parent groups only, analyses were repeated covarying for age. Findings did not meaningfully change, and we therefore report results from models without any covariates.

#### Clinical-Behavioral Correlates (see Additional file 1: Table S3 for results)

Using Pearson correlations, potential clinical-behavioral correlates of gaze were examined in the ASD group using the ADOS, ADI-R, and The Reading the Mind in the Eyes Test (a measure of social cognition) [87]. No significant associations were found, and data are reported in Additional file 1: Table S3.

## Results

Below we include statistical details for significant findings for probands and parents only (*p* < 0.05), with detailed significant and non-significant findings reported in Tables 3, 4, 5, 6). All BAP findings are reported in detail below.

**Table 3:** Summary of results—ASD versus control groups

	Control group	ASD group	Group differences						
	*M* (SD)	*M* (SD)	*F*	*Pillai's Trace*	*df*	*p*	*padj*	partial *η*^2^	Cohen's *d*
**Standard Gaze variables**									
(1) Dwell time (%)—social	75.54 (12.41)	70.37 (16.21)	1.40	0.05	2, 53	0.255	0.592	0.050	0.452
(1) Dwell time (%)—non-social	14.54 (7.32)	19.03 (12.63)							
(2) Fixation count (%)—social	71.25 (12.63)	67.46 (15.74)	0.65	0.02	2, 53	0.524	0.755	0.024	0.309
(2) Fixation count (%)—non-social	17.85 (7.98)	21.00 (13.42)							
**Fixation patterns**									
(4) Perseverative fixations (%)—social*	74.18 (30.88)	65.05 (35.51)	**3.27**	**0.11**	**2, 53**	**0.046**	0.149	**0.110**	**0.693**
(4) Perseverative fixations (%)—non-social*	7.08 (14.94)	23.40 (32.13)							
(5) Regressive fixations (%)—social	71.01 (12.43)	67.22 (16.43)	0.62	0.02	2, 53	0.541	0.755	0.023	0.303
(5) Regressive fixations (%)—non-social	18.09 (8.77)	21.30 (13.54)							
(6) Fixation transition									
Social to social (%)	64.86 (17.87)	59.48 (23.70)	0.98	0.053	3, 52	0.410	0.755	0.053	0.467
Non-social to non-social (%)	4.26 (6.59)	7.44 (7.87)							
Social to non-social (%)	15.71 (9.50)	16.43 (9.10)							
Non-social to social (%)	15.18 (6.52)	16.65 (10.24)							
**First fixations**						*FET*			
(7) First fixation AOI (%)—social	85.70	90.50	–	–	–	0.688	1.261	–	–
(7) First fixation AOI (%)—non-social	14.30	9.50	–	–	–			–	–
(8) First fixation duration (s)	273 (160)	240 (107)	0.79	–	1, 54	0.377	0.592	0.014	0.235
**Distribution patterns**									
(9) Fixation rate (exploration)	3.34 (.56)	3.52 (.60)	1.35	–	1, 54	0.251	0.345	0.024	0.309
(10) Fixation rate (exploration) AOI (fix/s)—social	3.10 (0.59)	3.37 (0.7)	1.51	0.06	2, 53	0.231	0.282	0.055	0.476
(10) Fixation rate (exploration) AOI (fix/s)—non-social	4.33 (1.05)	4.09 (1.47)							
(11) Fixation spatial distribution/coverage—5 × 4 (larger)	35.32 (5.29)	35.97 (6.62)	0.17	–	1, 54	0.686	0.755	0.003	0.108
(11) Fixation spatial distribution/coverage—10 × 8 (smaller)	58.52 (8.84)	62.92 (13.59)	2.14	–	1, 54	0.149	0.149	0.038	0.392

Bold values indicate significance at *p* < 0.05; *Nonparametric Mann–Whitney *U* results are presented in the body of the manuscript; *FET* = Fisher's Exact Test; *padj* reflects the Benjamini–Hochberg correction at a false discovery rate of .10

**Table 4:** Summary of Results—ASD parent versus control parent groups

	Control parent group	ASD parent group	Group differences						
	*M* (SD)	*M* (SD)	*F*	*Pillai's Trace*	*df*	*p*	*padj*	partial *η*^2^	Cohen's *d*
**Standard Gaze variables**									
(1) Dwell time (%)—social	71.57 (13.95)	65.5 (11.67)	2.80	0.06	2, 97	0.066	0.097	0.055	0.479
(1) Dwell time (%)—non-social	18.87 (10.05)	22.36 (8.76)							
(2) Fixation count (%)—social	68.17 (13.27)	63.71 (10.24)	1.98	0.04	2, 97	0.144	0.144	0.039	0.400
(2) Fixation count (%)—non-social	21.28 (9.33)	24.38 (8.46)							
**Fixation patterns**									
(4) Perseverative fixations (%)—social*	72.83 (30.64)	62.8 (33.78)	1.50	0.03	2, 97	0.228	0.279	0.030	0.349
(4) Perseverative fixations (%)—non-social*	17.89 (23.93)	18.00 (22.19)							
(5) Regressive fixations (%)—social	68.50 (13.69)	63.44 (10.44)	2.31	0.05	2, 97	0.104	0.143	0.046	0.436
(5) Regressive fixations (%)—non-social	21.50 (9.61)	24.80 (8.92)							
(6) Fixation transition									
Social to social (%)	58.37 (16.46)	53.45 (14.13)	1.02	0.031	3, 96	0.387	1.194	0.031	0.355
Non-social to non-social (%)	6.84 (7.77)	8.95 (7.95)							
Social to non-social (%)	17.70 (7.42)	18.75 (7.32)							
Non-social to social (%)	17.09 (7.52)	18.85 (7.28)							
**First fixations**						*FET*			
(7) First fixation AOI (%)—social	77.40	80.40	–	–	–	0.784	1.232	–	–
(7) First fixation AOI (%)—non-social	22.60	19.60	–	–	–			–	–
(8) First fixation duration (s)	222 (122)	259 (147)	1.73	–	1, 98	0.192	0.279	0.017	0.261
**Distribution patterns**									
(9) Fixation rate (exploration)	3.43 (0.55)	3.43 (0.60)	0.00	–	1, 98	0.981	5.396	0.000	0.000
(10) Fixation rate (exploration) AOI (fix/s)—social	3.25 (0.64)	3.34 (0.68)	0.84	0.02	2, 97	0.434	1.194	0.017	0.261
(10) Fixation rate (exploration) AOI (fix/s)—non-social	4.08 (0.96)	3.86 (1.13)							
(11) Fixation spatial distribution/coverage—5 × 4 (larger)	34.49 (5.80)	36.75 (6.77)	2.96	–	1, 98	0.088	0.097	0.029	0.109
(11) Fixation spatial distribution/coverage—10 × 8 (smaller)	57.68 (8.93)	60.49 (8.82)	2.39	–	1, 98	0.126	0.144	0.024	0.394

*Nonparametric Mann–Whitney *U* results are presented in the body of the manuscript; *FET* = Fisher's Exact Test; padj reflects the Benjamini–Hochberg correction at a false discovery rate of .10

**Table 5:** Summary of results for temporal dynamics GCA analyses—percentage of fixations over time

	Control > ASD											
	Estimate	*t*	*p*	Cohen's *d*								
Intercept	− **0.74**	− **30.42**	**< 0.0001**	− **8.209**								
Linear	− 0.10	− 0.75	0.453	− 0.203								
Quadratic	− 0.21	− 1.58	0.120	− 0.427								
Cubic	− 0.01	− 0.05	0.962	− 0.014								

	Control parent > ASD parent											
	Estimate	*t*	*p*	Cohen's *d*								
Intercept	− **0.72**	− **44.73**	**< 0.0001**	− **9.171**								
Linear	0.16	1.53	0.126	1.53								
Quadratic	0.10	0.92	0.360	0.189								
Cubic	**0.23**	**2.24**	**0.025**	**0.459**								

	Control parent > BAP (+)				Control parent > BAP(-)				BAP(-) > BAP (+)			
	Estimate	*t*	*p*	Cohen's *d*	Estimate	*t*	*p*	Cohen's *d*	Estimate	*t*	*p*	Cohen's *d*
Intercept	− **0.08**	− **0.26**	**0.010**	− **0.067**	− 0.15	− 0.54	0.590	− 0.124	**− 0.07**	**− 2.08**	**0.038**	**− 0.545**
Linear	**0.44**	**3.31**	**0.001**	**0.859**	− 0.02	− 0.16	0.876	− 0.037	**0.46**	**3.38**	**0.001**	**0.886**
Quadratic	**0.39**	**2.91**	**0.004**	**0.755**	− 0.09	− 0.76	0.446	− 0.174	**0.48**	**3.50**	**0.001**	**0.917**
Cubic	**0.42**	**3.20**	**0.001**	**0.830**	0.11	0.10	0.320	0.023	**0.30**	**2.28**	**0.023**	**0.598**

Bold values indicate significance at *p* < 0.05

**Table 6:** Summary of Results—Group differences in factor scores derived from the PCA

	M (SD)	M (SD)	*F*	*df*	*p*	*padj*	partial *η*^2^	Cohen's *d*
	**Control group**	**ASD group**						
Social/non-social attention (factor 1)	0.45 (0.88)	− 0.02 (1.38)	2.39	1, 53	0.128	0.085	0.043	0.418
Efficiency of exploration (factor 2)	0.08 (1.01)	0.01 (1.12)	0.06	1, 53	0.818	0.149	0.001	0.062
	**Control parent group**	**ASD parent group**						
Social/non-social attention (factor 1)	0.10 (1.01)	− 0.29 (0.81)	**4.40**	**1, 95**	**0.039**	**0.026**	**0.044**	**0.426**
Efficiency of exploration (factor 2)	− 0.08 (0.85)	0.04 (1.06)	0.33	1, 95	0.568	0.142	0.003	0.109

Bold values indicate significance at *p* < 0.05; padj reflects the Benjamini–Hochberg correction at a false discovery rate of .10

### Standard gaze variables (Fig. 3)

Findings from the standard gaze variables have been previously reported with different statistical analyses [23] and are reported below for the purpose of comparison to non-standard analytic methods.

**Fig. 3:**
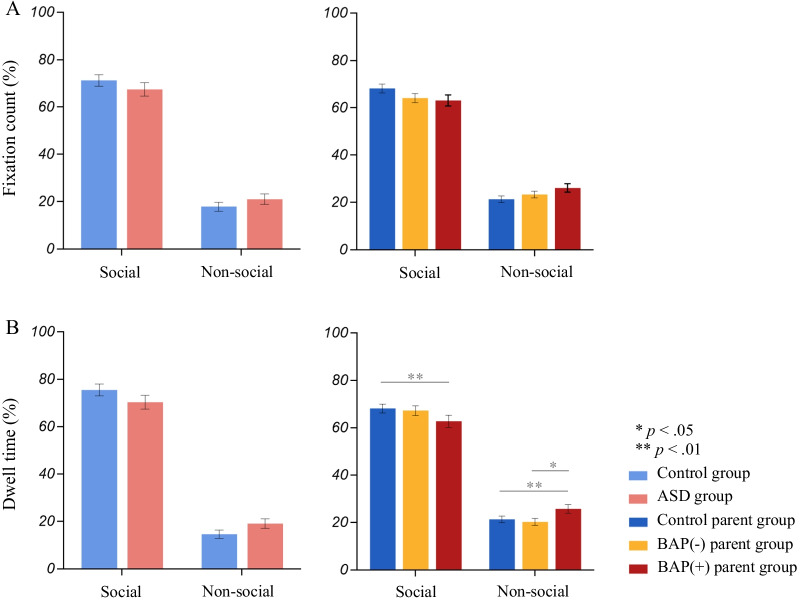
Overall gaze variables depicting **A** fixation count and **B** dwell time. Significant differences between BAP(+) and Control parent groups emerged in dwell time across social and non-social visual attention

**(1)** **Dwell time**:

*Probands and Parents*. There were no significant differences between the ASD and control group, or between parent groups in the percentage of time spent attending towards social or non-social information.

*BAP*. On average, the overall model for social and non-social looking times was significant across BAP(+), BAP(−), and control parents (*F*_(4,197)_ = 2.71, Pillai’s Trace = 0.11, *p* < 0.05, partial *η*^2^ = 0.053), with significant differences emerging in both social (*F*_(2,97)_ = 3.70, *p* < 0.05, partial *η*^2^ = 0.071) and non-social (*F*_(2,97)_ = 4.44, *p* < 0.05, partial *η*^2^ = 0.084) looking time. Pairwise comparisons revealed that BAP(+) parents on average showed significantly reduced social looking compared to the control parent group (mean difference = − 8.77, *p* < 0.01) and increased non-social looking compared to both BAP(−) parents (mean difference = 5.51, *p* < 0.05) and control parents (mean difference = 6.84, *p* < 0.05).

**(2) Fixation count**:

*Probands and Parents.* No significant group differences emerged in the percentage of fixations directed towards social or non-social information.

*BAP*. No significant differences in BAP status emerged overall for social or non-social looking (*F*_(4,194)_ = 1.42, Pillai’s Trace = 0.06, *p* = 0.229, partial *η*^2^ = 0.028).

### Temporal dynamics (Fig. 4)

**Fig. 4:**
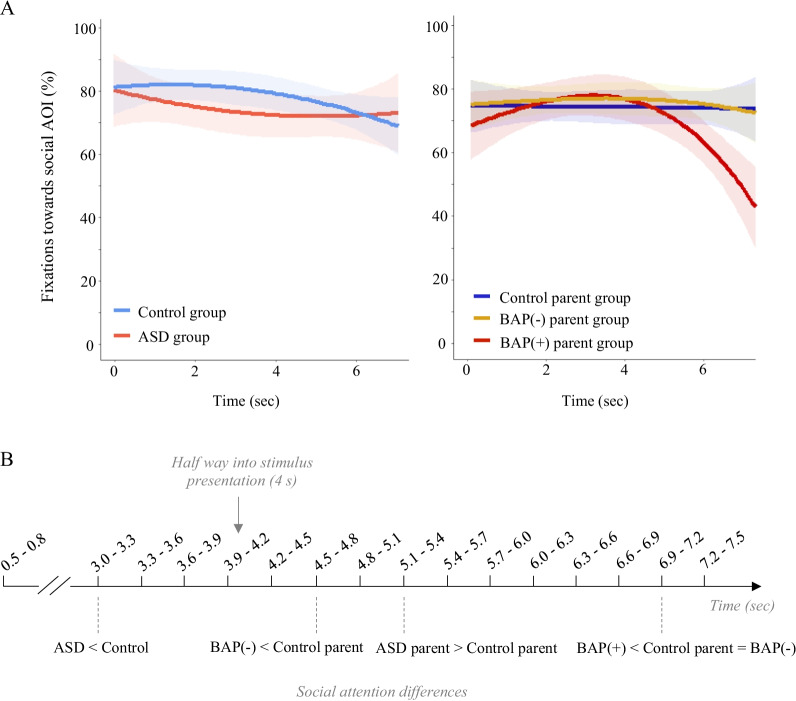
Dwell time patterns depicting **A** proportion of fixations over time (higher value indicates greater social attention) and **B** a schematic representing divergence time-bin analyses, where individuals with ASD were observed to attend less to social information than the control group half way into the stimulus presentation. Both BAP(−) and BAP(+) parents showed a spike in social attention around 5 s, with the BAP(+) group showing a striking decrease in social attention towards the end of the stimulus presentation compared to BAP(−) and Control parent groups

**(3) Fixations over time** (Growth Curve Analyses; Table 5, Fig. 4):

*Probands*. There were no significant group differences detected across linear (*Estimate* = 0.10, *t*(832) = 0.75, *p* = 0.453), quadratic (*Estimate* = 0.21, *t*(832) = 1.58, *p* = 0.12), or cubic (*Estimate* = 0.006, *t*(832) = 0.05, *p* = 0.962) terms in social versus non-social looking patterns over the course of the stimulus presentation. Time-bin divergence analyses revealed significant differences occurring halfway through the stimulus presentation, where individuals with ASD on average demonstrated significantly reduced social looking between 3000 and 3300 ms compared to controls. These findings indicate that individuals with ASD disengage from social information early, decreasing social fixations over time, whereas the control group showed increased social looking initially, that also decreased over the course of the task.

*Parents.* In contrast to findings in the ASD group, a significant group difference was detected for the cubic polynomial term (*Estimate* = − 0.23, *t*(1474) = − 2.24, *p* < 0.05), indicating that the ASD parent group shifted away earlier from social AOIs, and demonstrated decreased attention to social information over time, compared to the control parent group. To examine the exact moments at which time points groups differed from one another, divergence analyses demonstrated that, on average, ASD parents demonstrated reduced social looking patterns primarily during the beginning (i.e., 300–600 ms) and end (i.e., 4500–4800 ms and 6900–7200 ms) of the stimulus presentation (see Fig. 4). Interestingly, ASD parents also demonstrated increased social looking relative to the control parent group between 5100 and 5400 ms, showing a dynamic pattern of an increase followed by a decrease in social looking towards the end of the stimulus presentation, compared to the control parent group.

*BAP*. Significant group differences were detected for the linear, quadratic, and cubic terms, indicating that BAP(+) parents shifted away from social stimuli earlier, and demonstrated decreased social attention over the course of the task compared to the BAP(−) (*linear estimate* = 0.46, *t*(1474) = 3.38, *p* < 0.001; *quadratic estimate* = 0.48, *t*(1474) = 3.50, *p* < 0.001, *cubic estimate* = 0.30, *t*(1474) = 2.28, *p* < 0.05) and control parent groups (*linear estimate* = 0.44, *t*(1474) = 3.31, *p* < 0.001; *quadratic estimate* = 0.39, *t*(1474) = 2.91, *p* < 0.001, *cubic estimate* = 0.42, *t*(1474) = 3.20, *p* = 0.001). BAP(−) and control parent groups did not differ from one another across any linear, quadratic, or cubic terms (*linear estimate* = − 0.02, *t*(1474) = − 0.16, *p* = 0.876; *quadratic estimate* = − 0.09, *t*(1474) = − 0.76, *p* = 0.446, *cubic estimate* = 0.11, *t*(1474) = 0.10, *p* = 0.320). Divergence tests showed that, on average, the BAP(+) group fixated more towards social information relative to the control parent group towards the second half of the stimulus presentation (i.e., 5100–5400 ms), and showed a stark decrease towards the end of the viewing window (i.e., 6900–7200 ms). Similarly, on average, the BAP(+) group showed significantly decreased social attention towards the end of the stimulus compared to the BAP(−) parent group between 6900 and 7200 ms. In contrast, the BAP(−) group showed reduced social attention towards the second half of the stimulus presentation (i.e., 4500–4800 ms), with a later (i.e., 5100–5400) increase in social attention relative to controls.

### Fixation patterns (Figs. 5, 6, 7)

**Fig. 5:**
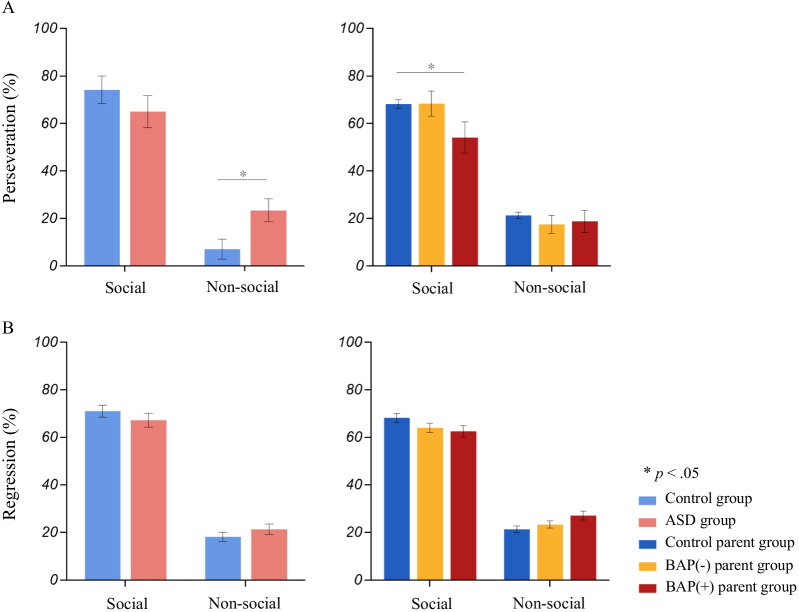
Fixation patterns depicting **A** Percentage of perseverative fixations and **B** Percentage of regressive fixations. Significant differences between ASD and control groups, and BAP(+) and Control parent groups emerged in perseverative fixation patterns, showing elevated non-social and reduced social perseverative fixations, respectively

**Fig. 6:**
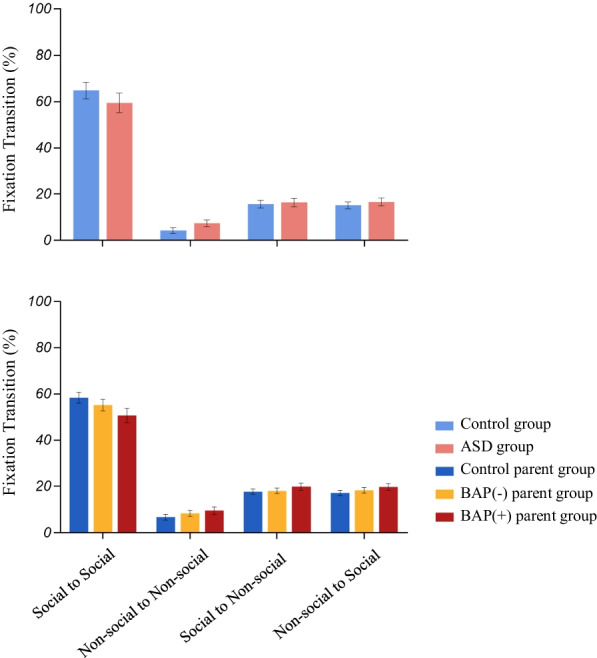
Fixation patterns depicting transition analysis (i.e., the percentage of fixations characterized as transitions from one AOI to another) as follows: social to social AOI, non-social to non-social AOI, social to non-social AOI, and non-social to social AOI transitions

**Fig. 7:**
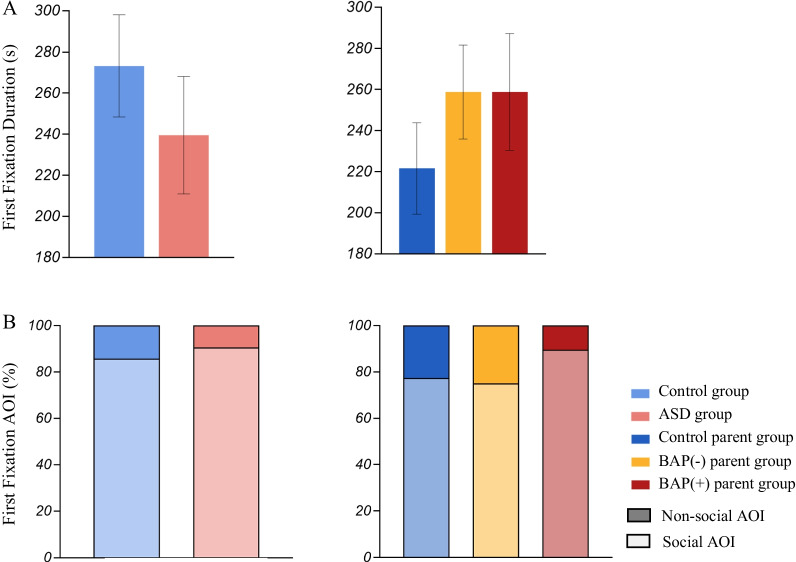
**A** First fixation duration, and **B** First fixation AOI showing both percentage of first fixations that were made towards social information (lighter shade) and non-social information (darker shade)

**(4) Perseverative fixations**:

*Probands*. MANOVA results revealed significant differences between ASD and control groups in the percentage of perseverative fixations towards social and non-social information (F_(2,53)_ = 3.27, Pillai’s Trace = 0.11, *p* < 0.05, partial η^2^ = 0.110). Follow-up univariate ANOVA tests revealed that on average the ASD group made a greater proportion of perseverations on non-social information compared to the control group (F_(1,54)_ = 6.43, *p* < 0.05, partial η^2^ = 0.106). Follow-up nonparametric analyses confirmed these findings, *ASD* M_rank_ = 32.98; *controls* M_rank_ = 25.14; *U* = 276.5, *p* < 0.05. No significant differences emerged between groups for social perseverations (*ASD* M_rank_ = 26.13, *Control* M_rank_ = 30.28, *U* = 327, *p* = 0.324).

*Parents*. No significant group differences were found between ASD parent and control parent groups (F_(2,97)_ = 1.50, Pillai’s Trace = 0.03, *p* = 0.228, partial η^2^ = 0.030). Nonparametric analyses confirmed these findings (social AOI: *ASD parent* M_rank_ = 47.01, *Control parent* M_rank_ = 55.96, *U* = 976.5, *p* = 0.122; non-social AOI: *ASD parent* M_rank_ = 50.97, *Control parent* M_rank_ = 49.77, *U* = 1161, *p* = 0.827).

*BAP*. MANOVA results showed no difference in social or non-social perseverative looking patterns between BAP(+), BAP(−), or control parent groups (F_(4,194)_ = 1.59, Pillai’s Trace = 0.06, *p* = 0.179, partial η^2^ = 0.032). However, nonparametric Mann–Whitney U test revealed that the BAP(+) group (M_rank_ = 26.31) made, on average, significantly fewer perseverations towards social information compared to the control parent group (M_rank_ = 35.50) (*U* = 331.5, *p* < 0.05).

**(5) Regressive fixations**:

*Probands and Parents*. No significant group differences emerged for social and non-social regressive fixations.

*BAP*. No significant differences in social and non-social regressive fixations emerged by BAP status (F_(4,194)_ = 1.87, Pillai’s Trace = 0.07, *p* = 0.118, partial η^2^ = 0.037).

**(6) Fixation transition analyses**:

*Probands and Parents*. There were no significant differences in the percentage of transitions occurring (regardless of direction of fixation transitions) between social and non-social information.

*BAP*. BAP status did not affect overall findings across specific transition patterns (F_(6,192)_ = 0.72, Pillai’s Trace = 0.04, *p* = 0.637, partial η^2^ = 0.022).

### First fixations

**(7) First fixation AOI** and **(8) First fixation duration**:

*Probands and Parents*. No significant group differences emerged in the first fixations towards social and non-social information, or in the duration of their first fixations.

*BAP*. Similarly, no differences emerged when considering BAP status in parents in the proportion of social (*BAP(*+*)* 21%, *BAP*(−) 29%, *control parent* 29%) and non-social (*BAP(*+*)* 2%, *BAP*(−) 10%, *Control parent* 9%) first fixations.

### Distribution patterns (Fig. 8)

**Fig. 8:**
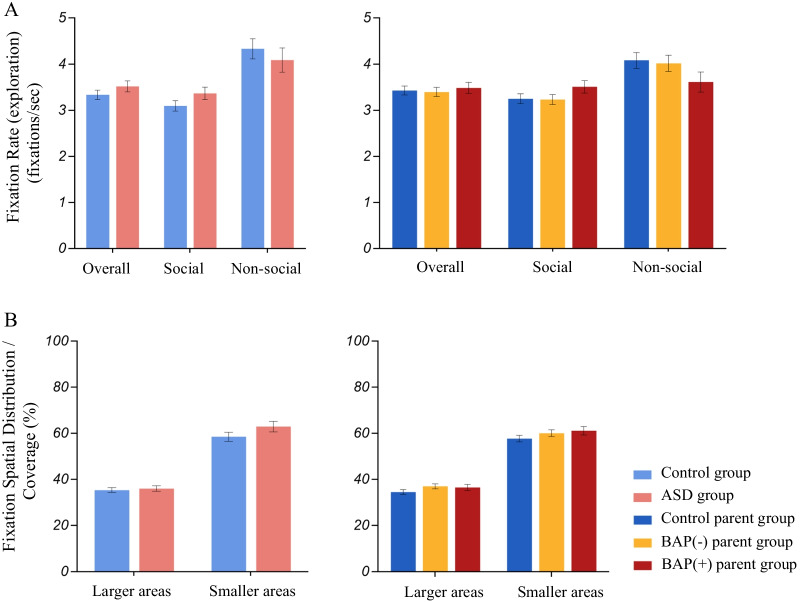
Distribution analyses depicting both A Fixation rate (exploration) overall and by AOI (social and non-social), and B fixation spatial distribution/coverage across larger (5 × 4) and smaller (10 × 8) boxes

**(9) Fixation rate (exploration), (10) Fixation rate (exploration) AOI, and (11) Fixation spatial distribution/coverage**:

*Probands and Parents*. Groups demonstrated a similar number of fixations per second of track time regardless of AOI, and in the number of fixations per second of track time that were made towards social and non-social information. There were also no significant group differences in the percentage of unique areas explored in the matrix for larger or smaller “boxes”.

*BAP*. There were no differences across BAP groups and controls for any variable examined (Fixation rate: *F*_(2,97)_ = 0.17, *p* = 0.848, partial *η*^2^ = 0.003; Fixation rate AOI: *F*_(4,194)_ = 1.794, Pillai’s Trace = 0.07, *p* = 0.132, partial *η*^2^ = 0.036; Fixation spatial distribution/coverage: large boxes—*F*_(2,97)_ = 1.52, *p* = 0.225, partial η^2^ = 0.030) or small boxes—*F*_(2,97)_ = 1.29, *p* = 0.281, partial *η*^2^ = 0.026).

### Principal Component Analysis (PCA)

**Fig. 9:**
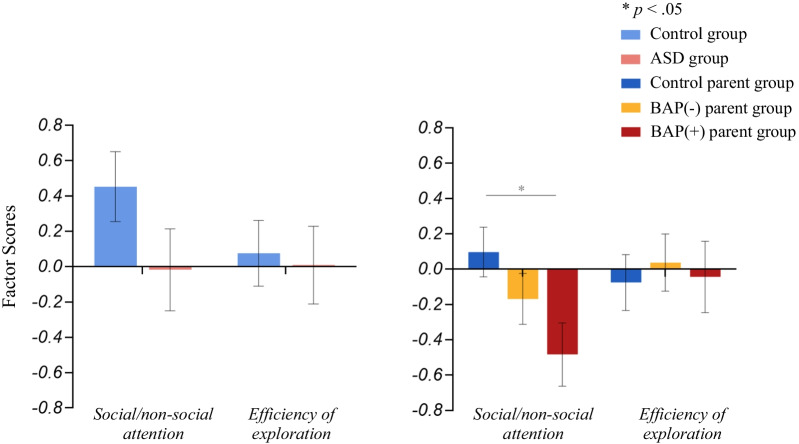
Group differences in factor scores from principal component analysis. Factor 1 = social/non-social attention (higher scores indicate greater social looking); factor 2 = efficiency of exploration

Examination of the scree plot (Additional file 1: Fig. S1) led to a two-component solution describing 58.6% of the variance of the data. The PCA yielded adequate loadings (≥ 0.30) on the two components (Additional file 1: Table S2). The first component consisted of all fixation variables tapping social versus non-social looking (henceforth, “social/non-social attention factor”. The second component consisted of fixation rates versus fixation spatial coverage efficiency—essentially reflecting the overall efficiency of spatial exploration in terms of fixation rates (henceforth, “efficiency of exploration”). For example, an individual scoring high on this component would have fixations that covered a relatively greater area, even if occurring less frequently; or conversely, fixations that were spatially confined even given an increased rate of fixation (Fig. 9).

*Probands.* There were no significant group differences between the ASD and control groups on the social/non-social attention factor (*F*_(1,53)_ = 2.39, *p* = 0.128, partial *η*^2^ = 0.043) or on the efficiency of exploration factor (*F*_(1,53)_ = 0.05, *p* = 0.818, partial *η*^2^ = 0.001).

*Parents.* The ASD parent group demonstrated significantly lower scores on average on the social/non-social attention factor (lower value indicating less social and more non-social looking) compared to the control parent group (*F*_(1,95)_ = 4.40, *p* < 0.05, partial *η*^2^ = 0.044). No differences in efficiency of exploration factor scores emerged between groups (*F*_(1,95)_ = 0.33, *p* = 0.568, partial *η*^2^ = 0.003).

*BAP.* Overall, there was a significant effect for the BAP (*F*_(2,97)_ = 3.29, *p* < 0.05, partial η^2^ = 0.064), with the BAP(+) group, on average, scoring significantly lower on the social/non-social attention factor relative to the control parent group (mean difference = 0.582, *p* = 0.01). No significant differences were found in efficiency of exploration factor scores by BAP status (*F*_(2,97)_ = 0.13, *p* = 0.880, partial η^2^ = 0.003).

## Discussion

This study applied a suite of eye-tracking analyses in attempt to deeply characterize potential differences in visual attention patterns to social and non-social information in ASD and the broad autism phenotype (BAP). Relatively robust differences were observed in patterns of fixations over the course of the stimulus presentation in the ASD and BAP parent groups, where both groups decreased social attention over time. Both the ASD and BAP(+) parent groups also showed differences in repeat perseverative fixations, with increased non-social and decreased social visual perseveration, respectively. Notably, although additional eye tracking analyses revealed relatively few differences in ASD and parent groups (regardless of BAP status), reducing data through principal component analysis revealed more robust group differences among parents, and these appeared largely driven by the subgroup showing the BAP. These findings underscore the utility of applying a broad array of analytic approaches to capture what may be nuanced, but still highly meaningful differences in visual attention in ASD and clinically unaffected relatives.

As noted, standard gaze variables (i.e., percentage of fixation duration and percentage of fixations) were re-produced from prior work [23] for comparison with the more expansive battery of variables examined here, and showed that parents with BAP traits spent more time generally fixating on non-social information compared to both parents without BAP features and control parents. Individuals with ASD in prior work showed similar trending patterns [23]. Gaze analyses beyond these standard variables were unique to the present study, with growth curve analyses examining changes in visual attention over time emerging as a primary variable distinguishing the ASD and BAP(+) groups from controls and BAP(−) parents. For these groups, fixations towards social information changed over the course of the task, wherein ASD and BAP(+) groups showed distinct fixation patterns from controls. Parents with the BAP shifted away earlier from social information, and showed decreased social attention over time compared to parents without the BAP and control parents. While this overall pattern was not significantly different in the ASD group, examination of the data revealed qualitatively similar fixation patterns over the course of the stimulus presentation in ASD and the BAP. Importantly, divergence analyses, performed to determine the exact moments in which groups diverged from each other, demonstrated that individuals with ASD showed significantly reduced social attention towards the middle of the stimulus presentation compared to controls, while BAP(+) parents showed a later divergence from control parents, occurring towards the second half, and an even more dramatic decline in attention to social AOIs in the final seconds of the stimulus presentation, compared to both control parents and BAP(−) parents. These findings converge with prior literature examining temporal dynamics of social attention among adults with ASD relative to controls [61, 62]. Both studies found a decrease of social attention among adults with ASD relative to controls, who instead looked away from social information initially but returned their attention back to social information towards the end of the stimulus presentation. Findings therefore provide additional evidence of divergent social attention temporal dynamics in ASD. Parallel findings from parents in the present study further suggest that these patterns may stem from genetic origins. Clinically, in light of Hedger and Chakrabarti’s (2021) findings that showed strengthening relationships between ASD traits (as measured by the Autism Quotient) and social attention over time, our findings support those authors’ conclusions that individuals with ASD demonstrate disparate sustained maintenance of social attention as opposed to initial orienting differences [62]. The lack of differences in first fixations AOIs in the present study provides further evidence for this conclusion. Moreover, because decreased social attention over time is believed to indicate a reduced interest for social stimuli [88], there may exist a diminished value or reward in social information among individuals with ASD and the BAP [62]. A reduction in the reward mechanisms associated with social information may potentially underlie the decay in social attention observed among our ASD and BAP (+) groups, and the subsequent increased interest towards non-social information (as reflected by perseverative fixations findings discussed below). It therefore appears necessary to examine timing effects in studies of visual perception and attention, particularly given prior work evidencing delayed global (i.e., gestalt or integrative) processing in non-social tasks in ASD [89] and atypicalities in the underlying neural correlates indexing face perception in later time windows [90, 91]. It is possible that in more dynamic stimuli involving prolonged looking, findings of decreased social attention may result in information overload [92]. Finally, results highlight how standard gaze variables may obscure shifting patterns of attentional engagement documented here, which may be meaningful to consider in understanding social-emotional processing differences in ASD. As such, an examination of fixations over the course of a stimulus presentation and divergence over specific time bins becomes an important step in further disentangling the nuanced and dynamic nature of gaze inherent to human behavior and impacted in ASD.

A prior study with this same sample, examining only standard eye-tracking variables [23] concluded that increased attention allocation towards non-social information in the TAT scene may reflect greater cognitive effort required to support narrative production (given the nature of the task); however, this pattern of visual attention did not improve narrative quality, suggesting that groups capitalized on different sources of information to inform their narratives. Patterns revealed by the more extensive gaze analyses performed in the current study suggest that these standard variables may not be sufficiently sensitive to capturing important shifts in attention during the narrative task. Specifically, increased attention towards non-social information over time suggests that *shifting* attention towards non-salient aspects of a scene may be advantageous in gleaning meaning from a scene to inform meaningful narratives. Given these findings, it is therefore possible that an examination of the second half of the scene (where individuals with ASD and ASD parents have already disengaged from social stimuli), may align more consistently with prior work that showed relationships between increased attention to non-salient information and greater clinical-behavioral impairments [40]. For example, individuals with ASD have been observed to demonstrate reduced attention towards the eye region of the face and more attention towards the mouth [17] and, during natural scenes in this same study, increased fixations towards non-social information was associated with poorer social adjustment and increased ASD symptom severity [17], complementing findings from a later study that showed relationships between elevated fixations directed towards the background of a scene that related to increased ASD symptom severity [36]. Future studies might benefit from a step-wise method of analysis that first includes an examination of fixations over the course of a task via GCA, followed by an application of divergence time bin analyses, and subsequent assessment of traditional and unique gaze analytical tools applied in the present study during critical time windows showing divergent patterns only. Together, that individuals with ASD and the BAP showed decreased fixations directed towards social information over time, suggests that this looking pattern may be particularly sensitive in reflecting ASD genetic vulnerability.

Other eye-tracking indices beyond standard fixation duration and proportion of fixations revealed further differences associate with ASD and the BAP. Specifically, individuals with ASD showed elevated rates of perseverative fixations towards non-social information and parents with the BAP showed reduced perseverations towards social AOIs. It may be that perseverative fixations reflect rigid tendencies, or a tendency to visually get “stuck” on certain visual information [55]. Prior work has also documented such patterns of perseveration among individuals with ASD or the BAP during eye-tracking tasks involving social and non-social images [28, 93], complex scenes [94], and language processing [27, 95]. Such perseverative tendencies are manifested behaviorally and are among the defining characteristics of ASD. They may also be evident in more subtle forms in the BAP [96–98], revealed through rigid or inflexible personality traits and behaviors reported in every-day life through both self-report questionnaires and objective, semi-structured interviews [49, 50, 66, 99–102].

Although relationships between gaze variables and clinical-behavioral features are not entirely consistent [17, 23, 36, 40], and the lack of such relationships in the context of this study as well (see Additional file 1: Table S3), observed patterns of refixations (i.e., perseverative or regressive fixations) have been shown to relate to both lower-order motoric RRBs and social communication in ASD [27, 28] and the BAP in parents [27]. A study examining gaze-language coordination additionally identified specific associations emerging between refixations in parents with the BAP and elevated rates of RRBs in their children [27]. Findings of atypical perseverative visual attention documented in ASD and the BAP in the present study may suggest that perseverative fixations or “sticky” visual attention could inform patterns of inheritance of ASD-related candidate endophenotypes. It additionally highlights the utility of examining eye-tracking data using methods beyond traditional fixation and duration eye-tracking variables.

Finally, it is possible that increased perseverative fixations towards non-social information in ASD and decreased preservative fixations towards social information may stem from differences in local (i.e., detailed) and global (i.e., integrative) visual processing in ASD more broadly [89, 103]. Individuals with ASD have been shown to demonstrate heightened local perceptual abilities (i.e., the enhanced perceptual functioning theory) which may result in a reduction in global perceptual abilities (i.e., weak central coherence theory), suggesting that they have difficulty shifting attention from local to a global level [104, 105]. Enhanced local perceptual abilities also may explain the tendency for individuals with ASD to more often focus on or get distracted by insignificant, non-salient details in their environment, which has been documented in both eye-tracking studies [23, 40] and autobiographical accounts (e.g., [106]). As such, it is possible that the findings documented in the present study contribute to both the social deficit and weak central coherence/enhanced perceptual functioning theories of ASD, which further necessitates studies examining the links between these theories.

In contrast to these relatively robust differences noted in perseverative fixations, there were few differences in proband and parent groups when applying other eye-tracking analytical techniques. Specifically, first fixation AOI or duration did not differ between groups. This was somewhat surprising, particularly in light of social orientation differences reported repeatedly in ASD [20, 37, 107]. It is possible that the demands of the task (i.e., providing a narrative after viewing the picture) influenced visual attention patterns in ways distinct from the passive viewing tasks used in much of prior work, where participants may explore the image as they please [28, 108]. Moreover, given that prior work has shown a lack of social orientation differences in higher functioning individuals with ASD (e.g., [109]), the higher cognitive ability of the sample included in the present study (i.e., verbal IQ > 80) may have further contributed to the null findings. First fixation methods may thus be more appropriate during infancy when social orientation comes online, applied to individuals who may have lower IQ, task methods involving passive viewing, and/or more dynamic stimuli.

Similarly, the lack of differences in fixation rate/exploration (i.e., number of fixations per second), transition, or fixation spatial distribution/fixation coverage analyses were surprising, given that prior work has documented differences in ASD, particularly during a face processing task [60]. As such, it may be that the concurrent narrative task demands necessitated both individuals with ASD and controls to explore the complex scene generously and comparably to deduce information to help inform their narratives; this stands in contrast to stimuli depicting motionless faces given their reduced “clutter” and visual complexity. Indeed, there is strong evidence in ASD demonstrating that patterns of fixations vary and depend highly on the context [40]. As such, use of fixation transitions, coverage, and exploration may be applied to future work examining visual attentional differences in the BAP during a face processing task, particularly given distinct face processing emerging in parents that have also been linked to underlying neural correlates [13].

Despite there being no group differences on most of these individual variables outlined above, when considered together, using data reduction methods to identify variables tapping similar constructs, they were fruitful in increasing power to detect subclinical differences associated with the BAP. Specifically, robust BAP-specific differences were observed on the social/non-social factor score compared to control parents, indicating that parents of individuals with the BAP looked significantly less at social information than ASD parents without the BAP or controls. Although significant group differences in scores of social/non-social looking were not identified between the ASD and control groups, the effect size of the difference was medium (and comparable to that of the parent groups). Of note, distribution measures that did not take social/non-social differences into account did not differentiate between parent or proband groups, underscoring the specificity of social communicative differences among ASD and the BAP, and providing further evidence that social attention differences may serve as a potential ASD-related endophenotype. These findings also emphasize the utility of extracting multiple eye-tracking outcome variables and synthesizing them through data reduction methodologies, such as PCA, such that the emergent properties associated with mechanistic underpinnings of special populations can be decoded. It is important to note that the results generated from PCA required the assembly of a large number of potential candidate eye-tracking markers associated with ASD risk, and that the interpretation and understanding of the specificity of the constructs required an in depth, independent examination of each marker’s specific performance and properties.

Analyses of clinical-behavioral correlates of gaze metrics in the ASD group revealed no significant associations (see Additional file 1: Table S3). These results are perhaps not surprising, given prior findings of inconsistent relationships between gaze variables and clinical-behavioral features [17, 23, 36, 40]. It is possible that although the measures of clinical-behavioral phenotypes examined (e.g., ADOS, ADI-R) are highly effective as diagnostic classifiers, they may not be sufficiently sensitive to capture more nuanced clinical variations related to eye-tracking metrics and associated underlying cognition, particularly in older participants without intellectual disability. It is also possible that associations may be observed between clinical scores and gaze variables at different developmental stages, or perhaps evident in samples comprised of individuals with a wider range of ability levels. Together these factors may limit our ability to capture individual clinical variation relevant to gaze metrics. Future studies may therefore consider including participants across the cognitive and age spectrum.

Based on recent convergent evidence demonstrating key timing effects in the social attentional profiles of ASD [61, 62], we emphasize the importance of applying growth curve and time-bin divergent analyses (or similar temporal models) in studies of gaze patterns. We highlight here key differences at various time points along the continuum of time that were obscured by overall metrics of gaze, which may similarly be important to examine in studies of gaze in other populations as well. Moreover, given prior literature documenting differences in perseverative fixations and their association with restricted and repetitive behaviors [27, 28], inclusion of perseverative fixations is important in the context of ASD. We additionally see the value added from the principal component analysis, which provided overarching context to the various gaze metrics included in this and prior literature, in addition to insights into the two components extracted from this type of analysis. Future work should aim to incorporate such granular methods (alongside broad-based metrics) to help elucidate nuanced trajectories of visual attention. These have direct implications for clinical translation, given that visual attention is indeed a dynamic process. An understanding of *when* and *how* an individual with ASD capitalizes on certain aspects of their visual world can provide keys into their underlying cognition and avenues for intervention. This work may serve to capture subtle phenotypic variability associated with social-communication impairments in these groups, and perhaps lead to more powerful, objective tools for monitoring treatment outcomes. Taken together, findings from this study may further our understanding of eye-tracking methods, their application to special populations and associated characterization of endophenotypes, and underlying cognitive and biological mechanisms contributing to ASD and the BAP.

## Limitations

Although adequately powered, larger sample sizes including groups with a wider range of verbal skills will be useful in future work, particularly for the principal component analysis. Additionally, although social attention differences have been repeatedly documented in ASD, such differences are not necessarily specific to ASD (though quality can differ; e.g., greater social anxiety levels were associated with faster orienting *away* from the eyes whereas elevated ASD traits were related to delayed orientation *towards* the eyes [110], and future work may consider including as additional control groups, populations not associated with ASD, in which social attention is impacted. Given emerging evidence for sex-related social attention differences [108, 111, 112], substantial evidence documenting social-communicative differences between males and females with ASD [113] and emerging evidence of sex-related differences in the BAP [114, 115], future studies should enrich for females to permit well-powered analyses of potential sex-specific patterns of the social attentional profiles examined here. This study also applied eye-tracking analyses to only one context (i.e., a complex scene depicting both social and non-social information). Future studies are warranted to further examine how these unique eye-tracking methods may be applied to studies of the BAP across stimuli varying in context. It is also important to acknowledge that the 11 methodological tools documented here, while applicable to research in ASD and subclinical features related to ASD, represent only a subset of a large number of analytic measures that can be applied to eye-tracking studies (e.g., see [116]).

## Conclusions

Taken together, this study highlights the specific utility of growth curve analyses to characterize meaningful fixation differences over the course of a task studies of visual social attention in ASD and the BAP. By providing a detailed examination of looking patterns over time, growth curve analyses may better capture the dynamic aspect of gaze that typically occurs in natural settings and which are impacted in ASD. In contrast, average dwell time and proportion of fixation variables assume a uniform or stagnant method of exploration, and tend to attenuate potential differences in looking patterns. Additionally, perseverations may be specifically tied to greater ASD risk, given differences observed in both ASD and the BAP. Despite most other variables yielding no robust differences between groups independently, when considered together using principal component analysis, this broad suite of eye-tracking variables contributed critical information to revealing relatively striking differences in social attention between the BAP and ASD from respective control groups. The eye-tracking variables examined in this study are thought to effectively reveal different aspects of underlying cognition [1, 9, 55–59, 79], and therefore may reveal key mechanistic insights into the roots of social functioning differences in ASD. These methods may additionally benefit studies of social skills training in individuals with ASD, as an objective means of measuring treatment outcomes. To our knowledge, this study is the most comprehensive application of different types of eye-tracking analytical methods in ASD and the BAP to date. Given the objective and measurable nature of the rigorous eye-tracking variables documented here, findings might contribute to development of a template that could be drawn upon in future eye-tracking studies examining visual attention across ASD and other populations.

## Supplementary Information

**Additional file 1**. Supplementary information provided outlining details pertaining to PCA analyses (**Fig. S1, Table S1,** and **Table S2**). **Table S3** displays a correlation matrix of the clinical-behavioral indices with eye-tracking variables among individuals with ASD.

## Data Availability

The datasets used and/or analyzed during the current study are available from the corresponding author on reasonable request.

## Abbreviations

AOI: Area of interest
ASD: Autism spectrum disorder
BAP: Broad autism phenotype
MANOVA: Multivariate analysis of variance
TAT: Thematic apperception test
