# Supplementary material for: A constellation of eye-tracking measures reveals social attention differences in ASD and the broad autism phenotype
Source: Mol Autism. 2022 May 4;13:18. doi: 10.1186/s13229-022-00490-w (PMC9069739; doi:10.1186/s13229-022-00490-w)
Supplement: Supplementary file 1 — Additional file 1. Supplementary information provided outlining details pertaining to PCA analyses (Fig. S1, Table S1, and Table S2). Table S3 displays a correlation matrix of the clinical-behavioral indices with eye-tracking variables among individuals with ASD. [file 13229_2022_490_MOESM1_ESM.docx]

**Supplementary Materials**

| *Supplementary Table 1: Pearson correlations between eye-tracking variables across groups included in PCA* | | | | | | | | | | | | | | | | | | | | | | | | | | | | | | | | | | | | | | |  |  |
| --- | --- | --- | --- | --- | --- | --- | --- | --- | --- | --- | --- | --- | --- | --- | --- | --- | --- | --- | --- | --- | --- | --- | --- | --- | --- | --- | --- | --- | --- | --- | --- | --- | --- | --- | --- | --- | --- | --- | --- | --- |
|  | |  | 1 | | 2 | | 3 | | 4 | | 5 | | 6 | | 7 | | 8 | | 9 | | 10 | | 11 | | 12 | | 13 | | 14 | | 15 | | 16 | | 17 | | 18 | | |  |
| Fixation count (%) - social | 1 | | | - | | -.67*** | | .92*** | | -.61*** | | .98*** | | -.66*** | | .63*** | | -.39*** | | -.51*** | | -.52*** | | .74*** | | -.63*** | | -.21** | | -.09 | | -.09 | | .07 | | -.15 | | .02 | | |
| Fixation count (%) - non-social | 2 | | | - | | - | | -.61*** | | .89*** | | -.66*** | | .98*** | | -.36*** | | .58*** | | .63*** | | .70*** | | -.91*** | | .70*** | | .20* | | .25** | | .04 | | -0.10 | | .07 | | .04 | | |
| Dwell time (%) - social | 3 | | | - | | - | | - | | -.70*** | | .92*** | | -.62*** | | .59*** | | -.39*** | | -.50*** | | -.45*** | | .69*** | | -.58*** | | -.17* | | -.06 | | -.31*** | | .26** | | -.21** | | .04 | | |
| Dwell time (%) - non-social | 4 | | | - | | - | | - | | - | | -.61*** | | .89*** | | -.36*** | | .56*** | | .57*** | | .58*** | | -.81*** | | .67*** | | .17* | | .20* | | .25** | | -.39*** | | .14. | | .00 | | |
| Regressive fixations (%) - social | 5 | | | - | | - | | - | | - | | - | | -.68*** | | .64*** | | -.39*** | | -.53*** | | -.51*** | | .74*** | | -.62*** | | -.22** | | -.08 | | -.11 | | .07 | | -.16* | | -.05 | | |
| Regressive fixations (%) - non-social | 6 | | | - | | - | | - | | - | | - | | - | | -.37*** | | .60*** | | .66*** | | .67*** | | -.91*** | | .71*** | | .22** | | .23** | | .08 | | .10 | | .11 | | .09 | | |
| Perseverative fixations (%) - social* | 7 | | | - | | - | | - | | - | | - | | - | | - | | -.54*** | | -.29*** | | -.25** | | .43*** | | -.42*** | | -.14. | | -.05 | | .02 | | .08 | | .03 | | -.11 | | |
| Perseverative fixations (%) - non-social* | 8 | | | - | | - | | - | | - | | - | | - | | - | | - | | .23** | | .20* | | -.47*** | | .63*** | | .12 | | .19* | | .10 | | -.09 | | .08 | | .03 | | |
| Transition: Social to non-social (%) | 9 | | | - | | - | | - | | - | | - | | - | | - | | - | | - | | .64*** | | -.83*** | | .19* | | .17* | | .07 | | -.02 | | -.15. | | -.04 | | .18* | | |
| Transition: Non-social to social (%) | 10 | | | - | | - | | - | | - | | - | | - | | - | | - | | - | | - | | -.81*** | | .17* | | .17* | | .14. | | -.08 | | -.05 | | -.04 | | .06 | | |
| Transition: Social to social (%) | 11 | | | - | | - | | - | | - | | - | | - | | - | | - | | - | | - | | - | | -.60*** | | -.25** | | -.19* | | -.00 | | .13 | | -.04 | | -.11 | | |
| Transition: Non-social to non-social (%) | 12 | | | - | | - | | - | | - | | - | | - | | - | | - | | - | | - | | - | | - | | .21** | | .22** | | .10 | | -.08 | | .16* | | .00 | | |
| Fixation spatial distribution/coverage - 5 x 4 (larger) | 13 | | | - | | - | | - | | - | | - | | - | | - | | - | | - | | - | | - | | - | | - | | .64*** | | -.31*** | | -.20* | | -.37*** | | .07 | | |
| Fixation spatial distribution/coverage - 10 x 8 (smaller) | 14 | | | - | | - | | - | | - | | - | | - | | - | | - | | - | | - | | - | | - | | - | | - | | -.20* | | -.14 | | -.20* | | -.01 | | |
| Fixation rate (exploration) AOI (fix/s) - social | 15 | | | - | | - | | - | | - | | - | | - | | - | | - | | - | | - | | - | | - | | - | | - | | - | | .10 | | .89*** | | -.22** | | |
| Fixation rate (exploration) AOI (fix/s) - non-social | 16 | | | - | | - | | - | | - | | - | | - | | - | | - | | - | | - | | - | | - | | - | | - | | - | | - | | .36*** | | -.07 | | |
| Fixation rate (exploration) | 17 | | | - | | - | | - | | - | | - | | - | | - | | - | | - | | - | | - | | - | | - | | - | | - | | - | | - | | -.20 | | |
| First fixation duration (s) | 18 | | | - | | - | | - | | - | | - | | - | | - | | - | | - | | - | | - | | - | | - | | - | | - | | - | | - | | - | | |
| Each cell contains the Pearson *r* and associated *p* values depicting correlations between each eye-tracking variable. **p* < .05, ***p* < .01, ****p* < .001 , *****p* < .0001. | | | | | | | | | | | | | | | | | | | | | | | | | | | | | | | | | | | | | | |  |  |

| *Supplementary Table 2: Component matrix from principal component analysis* | | |  |  |
| --- | --- | --- | --- | --- |
|  | Social/non-social attention (factor 1) | Efficiency of exploration (factor 2) | |  |
| Fixation count (%) - social | **0.88** | -0.10 | |  |
| Fixation count (%) - non-social | **-0.92** | -0.03 | |  |
| Dwell time (%) - social | **0.85** | -0.20 | |  |
| Dwell time (%) - non-social | **-0.88** | 0.07 | |  |
| Regressive fixations (%) - social | **0.88** | -0.10 | |  |
| Regressive fixations (%) - non-social | **-0.92** | -0.01 | |  |
| Perseverative fixations (%) - social* | **0.61** | 0.03 | |  |
| Perseverative fixations (%) - non-social* | **-0.60** | 0.05 | |  |
| Transition: Social to non-social (%) | **-0.68** | -0.14 | |  |
| Transition: Non-social to social (%) | **-0.68** | -0.15 | |  |
| Transition: Social to social (%) | **0.94** | 0.08 | |  |
| Transition: Non-social to non-social (%) | **-0.73** | 0.10 | |  |
| Fixation spatial distribution/coverage - 5 x 4 (larger) | -0.28 | **-0.63** | |  |
| Fixation spatial distribution/coverage - 10 x 8 (smaller) | -0.22 | **-0.50** | |  |
| Fixation rate (exploration) AOI (fix/s) - social | -0.11 | **0.86** | |  |
| Fixation rate (exploration) AOI (fix/s) - non-social | 0.19 | **0.37** | |  |
| Fixation rate (exploration) | -0.11 | **0.90** | |  |
| First fixation duration (s) | -0.06 | **-0.31** | |  |
| *Bold values indicate variables conceptualized under each component (factor) | |  | | |

| *Supplementary Table 3: Correlation matrix of PCA and clinical-behavioral indices among individuals with ASD* | | |
| --- | --- | --- |
|  | Social/non-social attention (factor 1); Pearson *r* | Efficiency of exploration (factor 2) Pearson *r* |
| ADOS Total Severity Score | 0.01 | 0.17 |
| SA Severity Score | -0.11 | 0.17 |
| RRB Severity Score | 0.26 | 0.10 |
| ADI-R Algorithm A (communication) | 0.20 | 0.24 |
| ADI-R Algorithm B (social) | 0.17 | 0.41 |
| ADI-R Algorithm C (RRB) | 0.14 | 0.06 |
| Reading the Mind in the Eyes (% correct) | -0.10 | -0.22 |
| ADI, Autism Diagnostic Interview-Revised; ADOS, Autism Diagnostic Observation Scale; RRB, Restricted and Repetitive Behaviors and Interests; SA, social-affect. | | |

***Supplementary Figure 1****. Scree plot from the principal component analysis. Arrow indicates the “elbow” determining 2 components.*


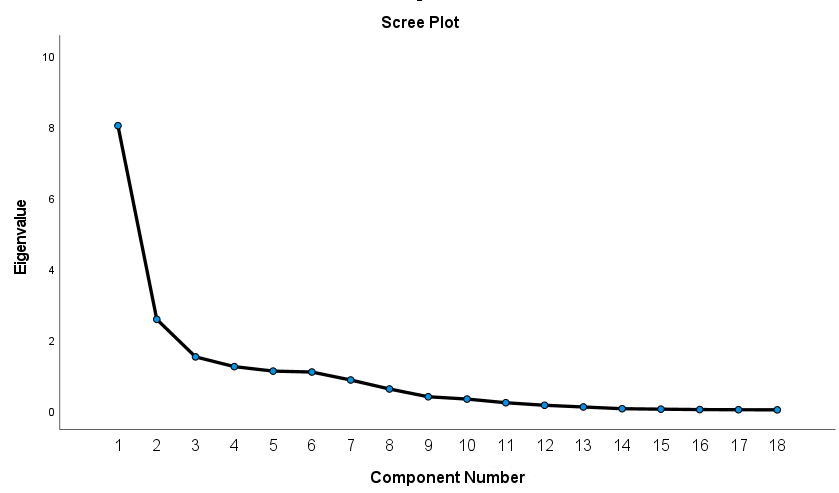


*Component Number*

*Eigenvalue*
